# Retinal burns from laser pointers: a risk in children with behavioural problems

**DOI:** 10.1038/s41433-018-0276-z

**Published:** 2018-12-13

**Authors:** E. Linton, A. Walkden, L. R. Steeples, A. Bhargava, C. Williams, C. Bailey, F. M. Quhill, S. P. Kelly

**Affiliations:** 1 Department of Ophthalmology, Bolton Hospitals NHS Foundation Trust, Bolton, UK; 2 Department of Ophthalmology, Lancashire Teaching Hospitals NHS Foundation Hospital, Preston, UK; 3 Bristol Eye Hospital, University Hospitals Bristol NHS Foundation Trust, Bristol, UK; 4 Department of Ophthalmology, Sheffield Teaching Hospitals NHS Foundation Trust, Sheffield, UK

## Abstract

**Objective:**

To explore self-inflicted retinal burns from laser pointers in children.

**Methods:**

Literature review of laser pointer retinal injuries in childhood and online survey of UK Consultant Ophthalmologists. A cohort of local children with self-inflicted injury is described. The matter is topical. We review progress in recent legislation and policy change in the UK.

**Results:**

Four of 77 case reports of laser burns in childhood analysed reported psychological or behavioural issues. Three of four children in our cohort had such issues. Delay in diagnosis occurred in two of our patients. Structural retinal damage persisted for over 12 months in all four children (seven eyes). Our survey of UK ophthalmologists found 159 cases of injury (85% male), 80% under 20 years of age. The majority of the laser pointers were purchased online. Many patients (36%) suffered moderate vision loss (6/18 to 6/60 Snellen), while 17% (at least 11 patients) suffered severe vision loss (<6/60 Snellen).

**Conclusion:**

We highlight the risk of macular damage and vision loss from handheld lasers specifically in children with behavioural, learning or mental health issues. The diagnosis may be difficult or delayed in such children. In children with uncertain macular changes, ophthalmologists should explore the history for possible instances of exposure to handheld lasers pointers. Regulatory authorities and manufacturers of handheld lasers need to be aware of the risk to children. Furthermore, there is a need to better inform parents, carers and teachers of the risk of ocular self-injury from such lasers pointers.

## Introduction

Laser pointers (sometimes termed laser pens) are handheld laser devices intended for pointing out objects or locations, including for demonstration and amusement purposes. Such lasers should have minimal risk of causing harm to vision. However, retinal injury from laser pointers is causing concern due to the wider availability of more powerful and cheaper laser pointers. The authors have encountered both adults and children with such injuries, including self-inflicted retinal injury from the misuse of high-powered handheld laser pointers. To explore this further in childhood we undertook both qualitative and quantitative (‘mixed methods’) research and also met with stakeholders in the UK. The material presented herein includes a literature review, a survey of UK ophthalmologists, clinical follow-up of seven eyes of four local children with self-inflicted laser burns diagnosed in the UK hospital practice and an update of our engagement with stakeholders and policy makers.

## Methods

### Literature review

We located all reports of laser pointer injury available on MEDLINE (on Ovid from 1966) and EMBASE (on Ovid from 1980) and ISI Web of Science (from 1990). Keywords and MESH terms for ‘laser pointer’ and ‘retina’ or its similes were used. The final list of titles and abstracts was screened by two reviewers (EL and AW) and full publications were obtained where articles were thought to be potentially relevant. Bibliographies of included studies and review papers were screened to identify other relevant studies. The literature search is accurate and up to date as of 19 March 2018. We searched for reports of self-inflicted laser burns where children were involved and then thematically explored for any psychological and behavioural features recorded in such reported childhood cases. We excluded patients described as being over 18 years old at the time of injury and studies where the full articles were not available in English.

### Survey of UK ophthalmologists

An online survey of UK consultant ophthalmologists was undertaken in January 2016 by one of our senior authors to explore their experience of laser pointer injury. A brief online survey was emailed to 990 consultant ophthalmologists in the UK, asking whether they had encountered a patient who suffered macular injury due to misuse of a handheld laser device.

Ophthalmologists who gave a positive answer were also asked: the number of such laser pointer burn patients they had encountered; ages and gender of patients; whether the injury was accidental, self-inflicted or deliberate; the power and colour of the laser beam and where purchased; visual outcome and optical coherence topography (OCT) and visual field evidence. To keep the survey brief and encourage completion, ophthalmologists who indicated seeing more than two patients were only asked to provide the details of the most and least affected patients. The data were analysed based on fully completed surveys.

### Case series

A convenience sample of four children (seven eyes) with self-inflicted retinal injury from laser pointers who presented to hospitals in Bolton, Bristol and Preston within a 12-month period and who have over 12 months follow-up are presented. Informed parental consent for publication of clinical details and images was obtained for all children in this cohort.

## Results

### Literature review

In the literature review we located 84 cases of handheld laser burns in children age 18 years or younger reported on 19 March 2018 [1–46]. (Table 1: Supplementary Material). This includes a case series that the senior authors (SPK and FMQ) previously provided [12]. Within these reports we systematically located one child with a pre-existing diagnosis of attention deficit hyperactivity disorder (ADHD), a second child had known learning difficulties and the third who was undergoing psychological treatment following a road traffic accident [8, 9, 17]. In one further case report we detected that a young person was referred for psychiatric evaluation following retinal injury from self-harming behaviour with handheld lasers [38]. We acknowledge a case report of laser maculopathy in a 20-year-old man in France with schizoid personality, but this is excluded from Table 1 that highlights cases of children [21]. Two further abstracts were identified of laser eye injuries in children but the full articles were not available in English; therefore, limited information is included in Table 1 and we cannot exclude any contributing psychological or behavioural problems in these children [22, 29].

**Table 1:** Literature review; 84 cases of handheld laser pointer burns in children aged 18 years or less.

Author (Year)	Country	Number of patients ≤ 18	Age	Gender	Mechanism	PMHx	Laser power	Clinical findings
Alsulaiman (2014) [3]	Saudi Arabia	11	17	Male	Inflicted by other	NS	NS	IS/OS disruption
			11	Male	Inflicted by other	NS	NS	Full-thickness macular hole, IS/OS disruption
			15	NS	NS	NS	NS	FTMH
			18	NS	NS	NS	NS	FTMH
			15	Male	Inflicted by other	NS	NS	Sub-ILM haemorrhage
			11	NS	NS	NS	NS	Subhyaloid haemorrhage
			18	NS	NS	NS	NS	Subhyaloid haemorrhage
			17	NS	NS	NS	NS	Subhyaloid haemorrhage
			17	NS	NS	NS	NS	Subhyaloid haemorrhage
			18	Male	Inflicted by other	NS	NS	ERM
			16	Male	Inflicted by other	NS	NS	Foveal cavity
Bhavsar (2015) [4]	USA	3	18	Female	Self-inflicted	Nil	50−100 mW	RPE changes, outer retinal hyper-reflective bands
			11	Male	Self-inflicted	Nil	NS	Disruption IS/OS and hyper-reflective bands
			14	Male	Inflicted by other	Nil	NS	Disruption IS/OS
Birtel (2017) [5]	Germany	7	12	5 Male, 2 Female	NS	NS	45 mW	Yellow lesion, pigment changes, IRF
			15		NS	NS	92 mW	Yellow lesion, pigment changes, SLOR
			15		NS	NS	65 mW	Pigment changes, SLOR
			9		NS	NS	95 mW	SLOR
			13		NS	NS	32 mW	SLOR
			15		NS	NS	NS	Pigment changes, SLOR
			13		NS	NS	NS	Pigment changes, SLOR
Dhoot (2014) [6]	USA	1	16	Male	Inflicted by other	Not stated	1000 mW	Macular hole
Dhrami-Gavazi (2015) [17]	USA	1	17	Male	Self-inflicted	ADHD	85−90 mW	Dense hyper-reflectivity outer retina
Dirani (2013) [7]	Lebanon	1	13	Male	Self-inflicted	NS	5 mW	Outer retinal disruption
Dolz-Marco (2016) [18]	USA	1	16	NS	Self-inflicted	NS	NS	Foveal pigmentary changes
Fujinami (2010) [8]	Japan	1	11	Male	Self-inflicted	Congenital Hearing Loss, Mental Retardation	NS	Highly reflective outer retinal mass
Hanson (2016) [9]	Switzerland	1	11	Female	Self-inflicted	Psychological Rx for RTA	<100 mW	Loss of photoreceptors and RPE
Israeli (2001) [22]	Israel	1	16	Male	Unknown	Unknown	Unknown	RPE disturbance
Lally (2014) [23]	USA	1	9	Male	Self-inflicted	NS	121 mW	Vertical hyper-reflective column in outer retina
Lee (2014) [2]	USA	3	6	Male	Self-inflicted	Nil	<5 mW	RPE disruption
			9	Male	Self-inflicted	Nil	121 mW	RPE disruption
			10	Male	Self-inflicted	Nil	<200 mW	Pigment clumping
Lim (2014) [24]	USA	1	13	Male	Self-inflicted	Nil	154 mW	Focal disruption and lucency of pigment epithelium
Mainster (2004) [10]	USA	5	11	Female	Self-inflicted	NS	NS	Foveal pigmentary changes
Noble (2015) [11]	USA	1	13	Male	Inflicted by other	Nil	50 mW	IS/OS disruption
Petrou (2014) [28]	UK	1	15	Male	Self-inflicted	NS	1000 mW	Bilateral macular holes
Pollithy (2012) [29]	Germany	1	11	Male	Self-inflicted	Unknown	55 mW	Unknown (Article in German)
Raoof (2014) [12]	UK	5	9	Male	Self-inflicted	NS	42−72 mW	Outer retinal layer disruption
			11	Male	Inflicted by other	NS	Unknown	Outer retinal layer disruption
			15	Female	Self-inflicted	NS	Unknown	Outer retinal layer disruption
			8	Male	Self-Inflicted	NS	Unknown	Outer retinal layer disruption
			13	Male	Self-inflicted	NS	Unknown	Fibrovascular membrane
Raoof (2016) [31]	UK	16	Range 9−16	12 Male	Not specified	NS	NS	11 children—focal retinal disruption in photoreceptor and ellipsoid layers.
			Mean 12.7	4 Female				2 children—diffuse disruption of outer retina
								3 children—subfoveal loss of outer retinal architecture and overlying hyper-reflective material in inner retina.
Rusu (2013) [32]	USA	1	15	Male	Unknown	Unknown	Unknown	Grey/yellow spots at the fovea showing hyperautofluorescence. OCT showed disruption of ELM, ellipsoid zone and interdigitation zone
Sanchez-Barahona (2016) [33]	Spain	1	9	Male	Self-inflicted	NS	NS	Hypopigmented lesion at fovea and vertical hyper-reflective bands on OCT
Sell (1999) [34]	USA	1	11	Female	Inflicted by other	NS	5 mW	Outer retinal layer disruption
Sethi (1999) [35]	UK	1	9	Female	Inflicted by other	NS	NS	Normal examination
Sheyman (2016) [37]	USA	1	15	Female	Self-inflicted (presumed)	Suspicion of psychological instability	Unknown	Profound IS/OS, interdigitation line and RPE loss in fovea and pigment migration
Simonett (2016) [38]	USA	1	17	Male	Self-inflicted	Nil but Dx with adjustment disorder & anxiety on psychiatric referral	45 mW	Outer nuclear layer hyper-reflectivity, disruption of IS/OS junction, full-thickness macular hole
Turaka (2012) [13]	USA	1	13	Male	Inflicted by other	Shaken baby syndrome	5 mW	Disruption RPE layer and non-specific thickening
Ueda (2011) [40]	Japan	1	13	Male	Inflicted by other	Nil	20 mW	Break in IS/OS junction and highly reflective region from RPE to ELM.
Weng (2015) [41]	USA	1	12	Male	Self-inflicted	Nil	100 mW	Focal loss of ellipsoid and interdigitation zones, irregularity of ELM and hyper-reflective area adjacent to RPE.
Wyrsch (2010) [43]	Switzerland	1	15	Male	Self-inflicted	NS	150 mW	Dense subretinal haemorrhages
Xu (2014) [14]	USA	1	12	Male	Self-inflicted	NS	48 mW	Damage to photoreceptors and RPE
Xu (2016) [44]	USA	4	12	Male	Self-inflicted	NS	NS	Disruption of IS-OS segment
			9	Male	Self-inflicted	NS	NS	Subfoveal outer retina hyper-reflectivity
			16	Male	Self-inflicted	NS	NS	Disruption of ellipsoid zone
			12	Male	Self-inflicted	NS	NS	Disruption of ellipsoid zone
Yiu (2014) [1]	USA	1	9	Male	Inflicted by other	N/S	1250 mW	Preretinal haemorrhages both eyes
Zhang (2016) [15]	USA	5	11	Male	Inflicted by other	Nil	Unknown	Hyper-reflectivity in photoreceptor and RPE layer
			13	Male	Inflicted by other	Nil	Unknown	Hyper-reflectivity in photoreceptor and RPE layer
			8	Female	Self-inflicted	Nil	Unknown	Foveal gap ellipsoid zone and outer segment layer
			10	Male	Self-Inflicted	Nil	Unknown	Foveal outer segment and ellipsoid discontinuities
			14	Male	Self-inflicted	Nil	Unknown	Hyper-reflectivity in photoreceptor and RPE layer
Ziahosseini (2010) [46]	UK	1	Teenager	Male	Self-inflicted	Nil	NS	RPE disturbance

*NS* not stated, *IS/OS* inner segment/outer segment, FTMH full-thickness macular hole, *RPE* retinal pigment epithelium, *ELM* external limiting membrane, *ERM* epiretinal membrane, *IRF* intraretinal fluid, *SLOR* structural loss of outer retina

### Survey of UK ophthalmologists

The survey submitted to 990 Consultant Ophthalmologists in the UK, using a ‘mailing’ database of email addresses of UK NHS Consultants, by one of the senior authors (FMQ) had a response rate of 15.5% and identified 159 cases of macular injury. Many injuries occurred within the year preceding the survey (54%) with most of the affected patients (80%) under 20 years of age or male (85%).

Most laser pointers were reported as having been purchased online. Many patients (36%) suffered moderate vision loss (6/18 to 6/60 Snellen), while 17% suffered severe vision loss (<6/60 Snellen). Visual acuity was not affected in 15% of cases.

Many of the injuries happened due to lack of awareness of the danger, and were either self-inflicted (35%) or caused by a third party (36%). There were no cases of assault reported. No relevant results on the colour of laser beam were provided. The power of known devices exceeded 50 mW in 33% of cases. The survey has been presented as a poster [47].

### Case series

We describe four local children (seven eyes) with self-inflicted retinal damage from handheld laser pointers with more than 12 months follow-up. All showed persistent outer retinal lamellar layer defects on spectral domain ocular coherence tomography (SD-OCT). Three children had a history of mental health or psychological challenges. All cases presented to our three hospitals within a 12-month period.

#### Case 1

**Fig. 1:**
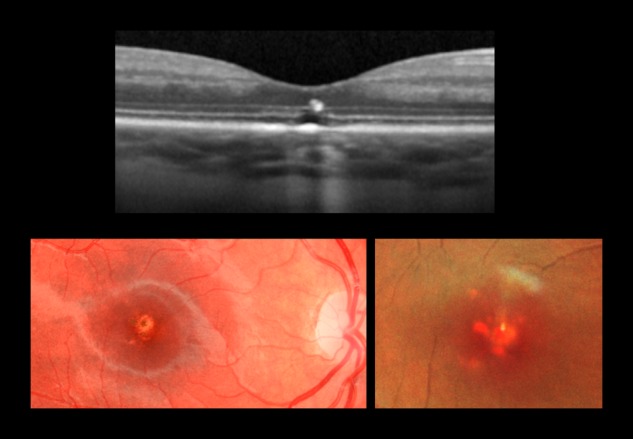
Patient 1: right eye. Baseline visit. Top panel: OCT image with outer lamellar layer defect. Colour fundus image lower left panel and multicolor image lower right panel.

An 11-year-old male, with a diagnosis of pathological demand avoidance (PDA) and migraine, presented to a community optometrist with a 2-day history of a black spot in the central vision of his right eye. The optometrist reported that the best corrected visual acuity (BCVA) in the right eye was reduced to 6/10 Snellen having been normal at a prior visit. Left was 6/5 Snellen. New pigmentary changes at both macula were observed by the optometrist and referral was made to the hospital eye service (HES). He was taking pizotifen prescribed for migraine. There was no relevant past ocular, medical or family history. Six weeks later, in the HES review, he described a persistent ‘blur’ in the central vision of the right eye. Unaided VAs were 6/9 right and 6/5 left. Pigmentary changes were noted at the central macula in both eyes. SD-OCT and imaging revealed bilateral outer lamellar layer defects (Fig. 1). Electrodiagnostic tests were normal but with limited co-operation. His mother accompanied him for all HES visits. In due course and following direct questioning he admitted to constructing a device made from Lego™ consisting of a laser pointer with a condensing lens used just prior to the onset of visual symptoms. The patient’s mother revealed that she had purchased the laser pointer online for him. During follow-up he reported symptomatic improvement and the VA remained stable. Centre involving structural defects at both macula persisted on clinical examination and OCT imaging to most recent follow-up 24 months later. (Supplementary Image 1). The mother confirmed the laser had been purchased from a well-known UK online retailer and was still available for online purchase a year following the incident.

#### Case 2

**Fig. 2:**
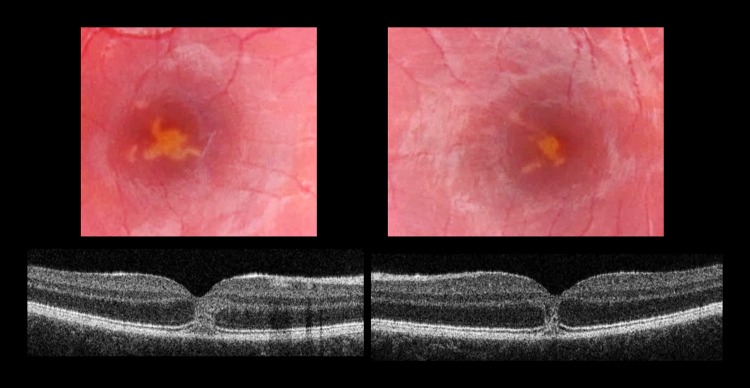
Patient 2. Baseline Visit. Top panel; Colour fundus photography showing yellow streak like lesions involving both fovea. Lower panel; OCT images both maculae show full-thickness hyper-reflective damage involving both fovea.

A 13-year-old male with attention deficit disorder (ADD) presented to the Emergency Medicine Department accompanied by his mother complaining of visual disturbance after staring into the beam from a toy laser for a few hours earlier that day. The patient stated that the toy laser belonged to a friend but the injuries were self-inflicted. The BCVA was 6/60 in the right eye improving to 6/36 with pinhole, and 6/12 in the left eye. SD-OCT images on presentation showed full-thickness hyper-reflective damage involving both fovea (Fig. 2). The patient was on methylphenidate 57 mg daily treatment for ADD and was known to Child and Adolescent Mental Health Services (CAMHS). He attended mainstream school with additional classroom support but was not classified as having special educational needs, with no statement of educational needs undertaken previously. Six weeks later, his BCVA had improved to 6/12 right and 6/9 left. An improvement in SD-OCT images was observed, notably an improvement in inner retinal layers. However, the centre involving outer lamellar layer defects on OCT and fundus changes persisted throughout 24 months of follow-up but decreased.

#### Case 3

**Fig. 3:**
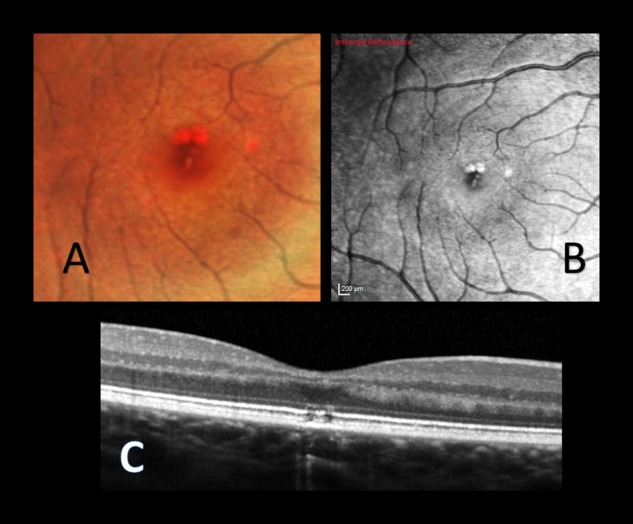
Patient 3. Right eye. Baseline Visit. (A) Multicolour fundus image and (B) infrared image showing discrete burns. (C) OCT image: outer lamellar layer defects seen.

A 15-year-old female with no past medical or psychological history was referred following a routine sight test where new discrete pigmentary changes at the right fovea were observed. The patient was asymptomatic. Her past ocular history, medical and family history were unremarkable. The unaided VA was 6/7.5 in both eyes. Two full-thickness centre involving round scars at the right fovea were observed and a third slightly eccentric. SD-OCT revealed defects in the ellipsoid zone in the outer retina in these lesions (Fig. 3). The patient admitted to being involved in a ‘competition game’ with three other children about 2 years previously in the home. The ‘game’ consisted of ascertaining which child could withstand a green laser beam in one eye for the longest time. She recalled shining the laser into her right eye for short duration, perhaps 10 s twice. The laser pointer had been purchased online by the patient’s mother.

The laser pointer responsible was retrieved from the family and sent for analysis. The analysis found the laser pointer was of wavelength 532 nm with an average power of 47 mW, making it a Class 3B laser. The label on the laser pointer incorrectly stated that it was 'Class II' with a maximum output less than 1 mW (Supplementary Image 2). At latest follow-up, at 24 months the macular changes persisted with 6/6 Snellen in each eye (Supplementary Image 3).

#### Case 4

**Fig. 4:**
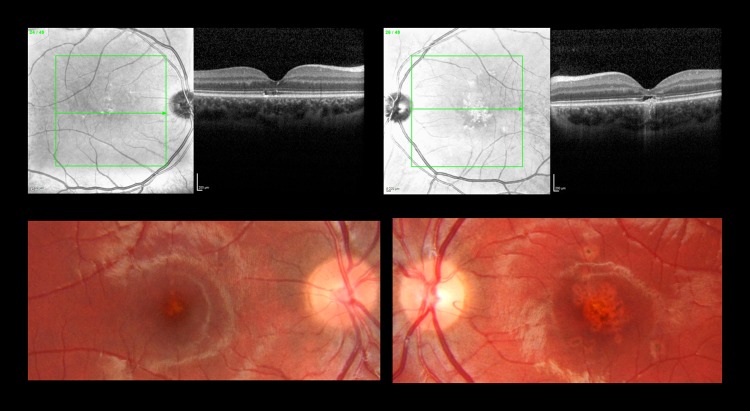
Patient 4. Top panels; Linked infrared and OCT images with outer lamellar layer. Lower panels; Bilateral multifocal macular pigmentary changes.

A 12-year-old boy was referred with a several month history of reduced vision in both eyes. He had a history of expressive and receptive language impairment and was attending a specialist school for children with cognitive impairment and disturbed behaviour. He was under CAMHS for anger and behavioural problems. There was no relevant past ocular or family history. The presenting BCVA were 6/30 right eye and 6/75 left eye. Colour vision was reduced, with only 4/17 Ishihara plates correctly identified in the right eye and 9/17 in the left eye. Bilateral multifocal macular pigmentary changes were noted (Fig. 4). To investigate abnormal visual function, electrodiagnostic tests and magnetic resonance imaging (MRI) of the brain and orbits were performed, both of which were normal.

At subsequent follow-up and on direct questioning, the boy revealed he had been playing with laser pointers at school, particularly in games involving looking directly into the beam of the laser pointer. His BCVA at 12 months follow-up was 6/19 right eye and 6/48 left eye. Fundus examination showed irregular pigmentation at the right macular and a scar at the left macular. OCT scan showed small, round, punched-out lesions more frequent in the left than the right macula.

## Discussion

Retinal burns from handheld laser pointers are an important and increasingly topical public health issue. Such devices are becoming more powerful, less costly, are often incorrectly labelled, and can be easily purchased online. Furthermore, there is increasing apprehension for aviation safety following suspected retinal injuries to commercial airline pilots falling victim to laser attacks [19, 27]. Lee et al. reported that young males were the most frequent group reported to sustain handheld laser pointer injuries in reports from 1999 to 2014 [2]. Our survey of UK ophthalmologists supports these findings, with results showing 85% of reported cases were male and 80% of all patients were under 20 years of age [47]. Our literature review also concurs with these findings, with 73% of cases being young males. While laser burns, including self-inflicted, can affect adults it is opined that children are at greater risk of laser pointer injuries than adults as they are intrigued by their appearance, and lack protective mechanisms of blinking and gaze aversion that adults exhibit and furthermore have clear ocular media which provides little protection from laser injury [2, 6, 7]. The majority of the laser pointer injury cases encountered by the UK ophthalmologists were reported as having occurred from laser pointers that had been purchased online. Clinical management of laser-induced retinal injuries is anecdotal, on occasion oral corticosteroids have been prescribed [2, 7, 13].

In our survey of UK ophthalmologists, the reported visual acuity in affected patients was reported as 6/18−6/60 in 36% and worse than 6/60 in 17% of cases [47]. In our literature review the visual acuity at presentation was 6/18−6/60 in 36% and worse than 6/60 in 28% of cases. The final visual acuity, where reported, was 6/18−6/60 Snellen acuity in 24% and worse than 6/60 in 5% of cases.

We acknowledge a recent review by Birtel et al. that identified 111 patients of unstated ages with laser pointer eye injuries in the literature [5]. They found highly variable retinal injuries across the literature, including macular holes, retinal haemorrhage and on OCT imaging disruption of retinal pigment epithelium, outer retinal hyper-reflectivity and disruption of outer retinal layers. That review did not document patient factors or patient age or if the injury was self-inflicted.

### Classifications and misclassification of lasers

The revised UK classification of laser products consists of eight categories: Class 1, 1C, 1M, 2, 2M, 3R, 3B and 4, with Class 4 lasers being the highest radiation hazard [48]. The World Health Organisation (WHO) stated in 1998 that “laser pointers higher than class 2 are considered too powerful for general use as laser pointers and present an unacceptable risk in the hands of consumers because they may cause eye injury” [49]. Class 2 laser products have a maximum power of 1 mW and fall within the visible wavelength range 400−700 nm. In 2014 Public Health England advised “the sale of laser products to the general public for use as laser pointers should be restricted to Class 1 or Class 2 devices” and further advised “toys should be class 1 or of such low output that they do not need to be classified” [50]. In the United States (US) the Food and Drug Administration (FDA) are: class I, IIa, II, IIIa, IIIb and IV with increasing numbers corresponding to higher output power [51]. FDA permits laser pointers with a maximum power of 5 mW (class IIIa) in the visible wavelength region of approximately 400−710 nm [52]. However, handheld laser pointers are widely available to purchase online, often do not conform to such regulations or carry appropriate labelling of the laser power or carry warnings with regard to the ocular risk involved. There are reports of these devices being misclassified and found to have a higher output than stated when objectively tested [2, 3, 9, 11]. Incorrect labelling increases ocular hazards; a consumer or parent may think that a Class 2 laser will be safe—but if in reality the device is a Class 3B then the risk will be far greater than anticipated. Recent publications have highlighted concerns of incorrect labelling of lasers in the USA, Australia and UK [53–55]. Case 3 in our series is a further example of misclassification. The parents of the children in our series reported that they were unaware of the ocular risks of children misusing laser pointers. This also chimes with other case reports. Lastly, in some cases the parents were unaware that their child was in possession of such devices [2].

### Classification and misclassification of laser retinal injury in children

Diagnosis of laser pointer retinal injuries in childhood can be difficult, as children and parents may be hesitant to admit to use and purchase of such devices. Additionally, laser retinal injuries may have similarities in clinical appearance to other retinal disorders and lead to misdiagnosis, delayed diagnosis and unnecessary investigations or treatment. A recent case series from Moorfields Eye Hospital reported that 5 of the 16 children with laser injury were initially suspected to have macular dystrophies which delayed their diagnosis [31]. Cases 1 and 4 in our series were also initially similarly mistaken as such. We are aware of another case locally being mistaken for macular inflammation. However, the changes seen on SD-OCT imaging namely focal disruption of the ellipsoid zone are diagnostic of photic maculopathy [2–4, 7, 9, 11, 13, 39]. The recognition of such outer retinal layer defects should prompt a thorough history to enquire if the child has been exposed to a beam from a laser pointer or sun gazing. Zhang et al. also commented on similarities between photic macular injuries and macular genetic conditions and opined laser pointer burns patients may improve over time whereas genetic conditions do not [15]. We noted some improvement in the seven eyes studied but all had centre involving structural damage on SD-OCT persisting after a year or more of follow-up. It has been opined that it may be possible to differentiate between self-inflicted and third-party-induced laser retinal injuries on SD-OCT imaging. Bhavsar et al. reported that self-inflicted laser injuries had a streak-like appearance, whereas injuries caused by others tended to be discrete lesions in close proximity to the fovea [4]. Our study does not confirm this impression as we saw discrete injury in the presence of self-inflicted injury. A recent report of four children suggested that the most significant variables predictive of retinal injury in laser pointers are the amount of energy delivered by the laser, duration of exposure and location of retinal involvement [44]. The Moorfields study of children added a proposed classification of severity of laser burn structural damage which we welcome [31].

### Behavioural and psychological issues in children with self-inflicted injury

Neither of two recent case series of childhood laser retinal burns or a recent literature review of cases of any age reported any children’s co-existent behavioural profiles or whether self-injurious behaviour (SIB) was a factor [5, 31, 44]. Similarly in the literature review we undertook searching for such themes in children; few reports gave details of children’s general or psychological status. We opine that many authors were either unaware of children’s behavioural issues or else did not report such details, including absence of any behavioural matters, in their case reports. In any event although we found a small number of case reports that noted existing psychological, psychiatric, behavioural or learning problems in those affected; to our knowledge no reports to date highlight the risk of handheld laser possession in such children or explore a relationship between these diagnoses and laser eye injuries [8, 9, 17, 38]. Case 1 in our series had a diagnosis of PDA syndrome. Newson et al. described children with PDA as having a resistance and avoidance of demands as well as impulsive and obsessive behaviour and suggested it be a clinical entity in its own right rather than a sub-type of autism [56]. The first systematic comparison of PDA and autism spectrum disorders in 2014 reported that children with PDA showed characteristics of both autism—such as peer problems—as well as traits of conduct disorders such as anti-social behaviour [57]. In our case 2, the patient had a diagnosis of ADD, also known as ADHD. Children with ADD/ADHD exhibit behavioural problems and inattention, hyperactivity or impulsivity [58]. In our opinion the common themes of impulsive, obsessive behaviours and a resistance to following instructions puts children with such conditions at risk of SIB and importantly more so if they are in possession or playing unsupervised with objects such as powerful handheld lasers. Our third child did not have any diagnosed mental health problems but did take part in a ‘game’ that exposed her to direct laser pointer exposure for whatever reason. Case 4 had complex behavioural challenges. Two of our four children were linked to CAMHS services.

Self-injurious behaviour (SIB) is considered “a class of behaviours, which the individual inflicts upon his/herself that have the potential to result in physical injury” [59]. Weiss explains the subtle comparison between individuals with developmental disabilities unknowingly behaving in a way that leads to harm, and those who set out with an intent to hurt themselves, for example in attempts to take their own life [60]. SIB has an estimated prevalence of 35−60% amongst people with autism [61]. Self-injurious trauma to the eye is recognised in children with autism and related conditions. Patton reviewed the relevant literature in 2004 and reported that ‘head-banging’ was a common mechanism of ocular injury in children with autism [62]. Very recently, Lee et al. reported three case of bilateral cataract following self-inflicted trauma in children with autistic spectrum disorder [63]. Our report highlights another novel ocular SIB in such individuals.

### Regulation of laser pointers

Recent editorials by Marshall et al. and Bartsch et al. provided perspectives on the regulation and safety and hazards of laser pointers from a UK and US viewpoint [26, 64]. The review by the Swedish Radiation Safety Authority of 46 cases from the world literature of laser pointer burns is useful as severity and mechanism of injury where known are outlined in that report [65]. In our clinical experience powerful handheld laser pointers in the hands of children with behavioural, learning and or mental health problems is a dangerous risk. We thus wish to draw addition to this hazard. The matter is relevant for parents and regulators. Importantly the classification of laser pointers in various jurisdictions and the advice by Public Health England in the UK does not take into account the potential for ocular harm from prolonged self-inflicted exposure, as occurred in the children reported herein. With regard to retinal hazards, labels seem designed for laboratory scientists and not necessarily for the general public and importantly labelling may not reflect the true class of the laser—as misclassified. A word such as “Class 3R” means little to the non-expert. The public may falsely assume that these ‘toys’ are safe as they are approved for general sale. Self-inflicted injury at close range in children and from misclassified laser pointers adds to our concern as does the increasing availability of cheaper and more powerful handheld lasers.

The senior authors (SPK and FMQ) have alerted the Royal College of Ophthalmologists and the Royal Society for the Prevention of Accidents on our concerns and attended a workshop on the matter hosted by Public Health England (PHE). The UK government reviewed the evidence of harm to children and risk to pilots following a multi-agency meeting in February 2016 before deciding its approach to tackling this mounting ocular public health problem. One of the senior authors (FMQ) used the data from the recent online survey of UK ophthalmologists to inform that multi-agency meeting [47]. Following this multi-agency meeting PHE launched an online health awareness video following the concern surrounding ocular hazards from laser pointers [66]. We welcome that video and publicity about laser pointers by some local trading standards authorities [67]. In May 2018, the Laser Misuse (Vehicles) Act gained Royal Assent. Under this new legislation individuals who target drivers of trains, buses, boats or planes can be jailed for up to 5 years, and the previous cap on the maximum fine of £2500 has been lifted [68]. The Government Department for Business, Energy and Industrial Strategy ran a Call for Evidence on Laser Pointers in 2017 and published their response in January 2018 [69]. This document summarises the four steps the Government will take in reaction to the call for evidence; provide additional support for enforcement activities around the import of high-powered lasers, encourage more effective voluntary labelling of laser pointers, promote public awareness on the hazards of laser pointers particularly eyesight and address pilots concerns via the aforementioned Laser Misuse (Vehicles) Act 2018 [68, 69]. We believe that this mixed methods publication assists in promoting awareness of a specific ocular public health concern in children in addition to the known wider concerns including for adults. Furthermore, the conviction and sentencing in 2016 of an individual for the sale of a laser pointer that caused eye injury in a child was an important step by UK authorities in the enforcement of the regulations surrounding the sale of laser products [70]. However, we remain concerned about online sale of powerful laser pointers.

The strengths of this mixed methods contribution include its addition to the public health debate and literature by highlighting the risks of retinal burns from laser pointers in children—particularly with respect to children with behavioural problems—and our engagement with UK laser safety stakeholders. We assessed the number, age and gender and visual outcomes of patients with laser injury encountered via UK consultant ophthalmic colleagues using an online survey. A limitation was the poor response rate and thus data so obtained do not provide the true incidence and clinical features of such cases; this which would require formal case finding such as the British Ophthalmic Surveillance Unit (BOSU) undertakes. Our case series is small but has over 12 months follow-up data. We are of the opinion that further formal public health case finding and surveillance research is warranted to assess the epidemiology of retinal laser pointer burns and the profile and outcomes of patients who sustain such injury. Cohort studies from hospital eye clinics would be of merit to provide information on OCT biomarkers and prognosis. Such matters may be complicated by the issues that parents may not be aware of their children having laser pointers and or families may be reluctant to disclose such information even where known.

## Implications for policy

The recognition by UK Government for the need for more robust regulation of the importation and sale of laser pointers, including online sales is reassuring as is the Laser Misuse (Vehicles) Act 2018 which was recently given Royal Assent [67]. There is a need for ophthalmologists to closely question all patients especially children with retinal outer lamellar layer defects (best appreciated on SD-OCT imaging) for any history of laser pointer exposure before considering further tests for macular disorders. Importantly there is a need for increased public awareness and education of the ocular hazards of laser pointers [69]. In particular, parents, and especially parents of children with conditions that may increase risk of self-injurious behaviour, should be aware that powerful and often incorrectly classified handheld lasers pointers can be dangerous to sight. Specifically, the availability of high powered and also mislabelled laser pointers remains a concern. Because such lasers are readily available, children likely to self-harm may be at a greater risk of shining laser beam into their eyes, perhaps for longer periods of time. We urge the manufacturers of handheld laser pointers and their vendors to consider our concerns. We urge the regulators, manufacturers and distributors of laser pointers—including online merchants—to be more vigilant given this novel concern of vision loss in at-risk children.

### Summary

#### What was known before

- Retinal burns from increasingly available and increasingly powerful handheld laser pointers are a mounting concern and of topical interest.

#### What this study adds

- We report that children with mental health, behavioural or learning difficulties are at risk from self-injury from such lasers and which may be mislabelled. Such retinal burns in children may be mistaken for other macular disorders. We outline policy changes afoot in UK.

## Electronic supplementary material

Supplementary image 1

Supplementary image 2

Supplementary image 3

LEGENDS FOR SUPPLEMENTARY FIGURES
