# Supplementary figures and images for: Retinal burns from laser pointers: a risk in children with behavioural problems
Source: Eye (Lond). 2018 Dec 13;33(3):492–504. doi: 10.1038/s41433-018-0276-z (PMC6460723; doi:10.1038/s41433-018-0276-z)

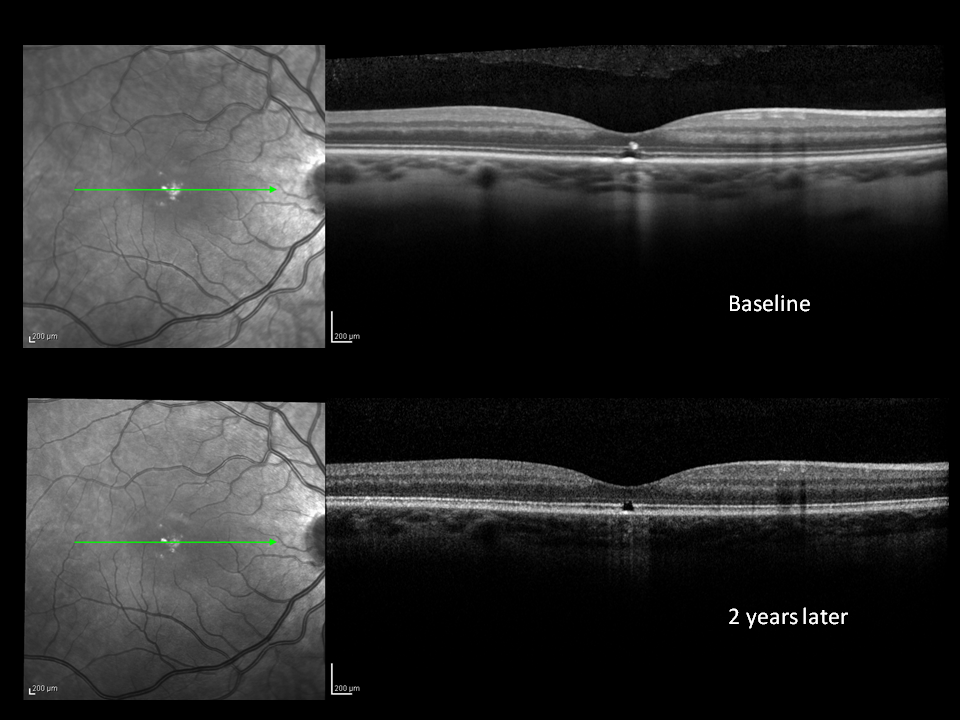

Supplement: Supplementary file 1 — Supplementary image 1 [file 41433_2018_276_MOESM1_ESM.tif]

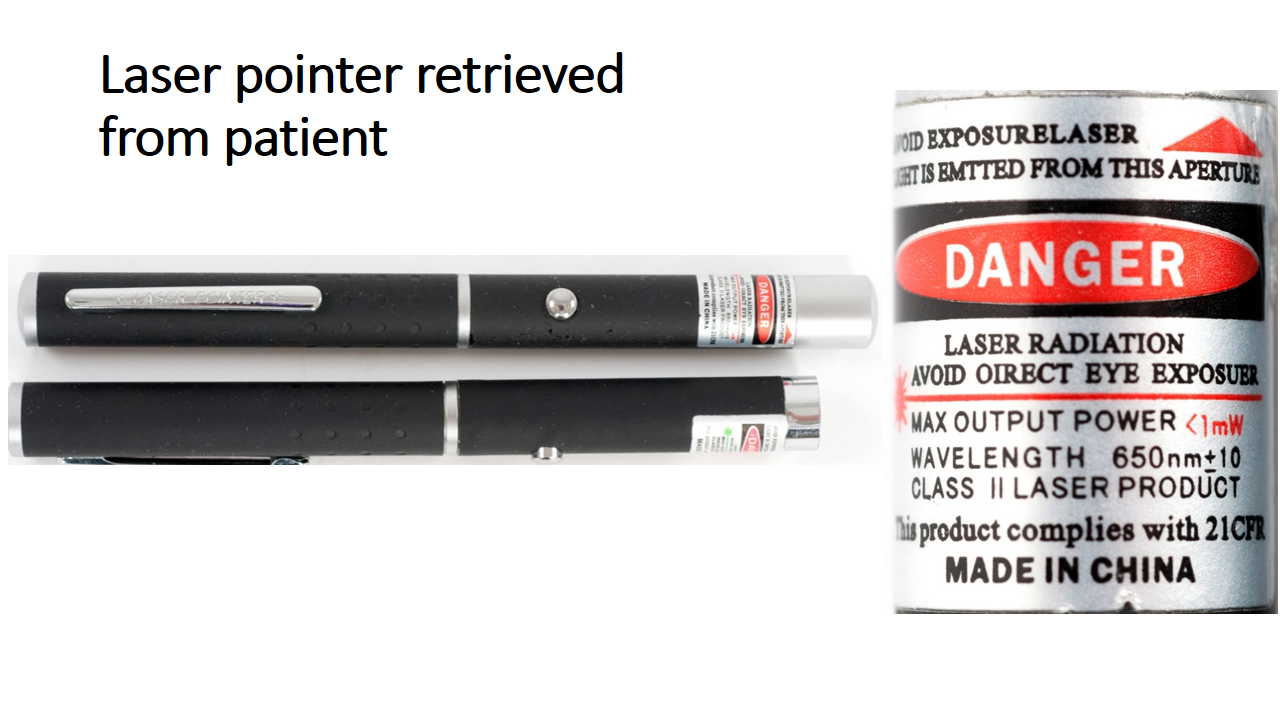

Supplement: Supplementary file 2 — Supplementary image 2 [file 41433_2018_276_MOESM2_ESM.tif]

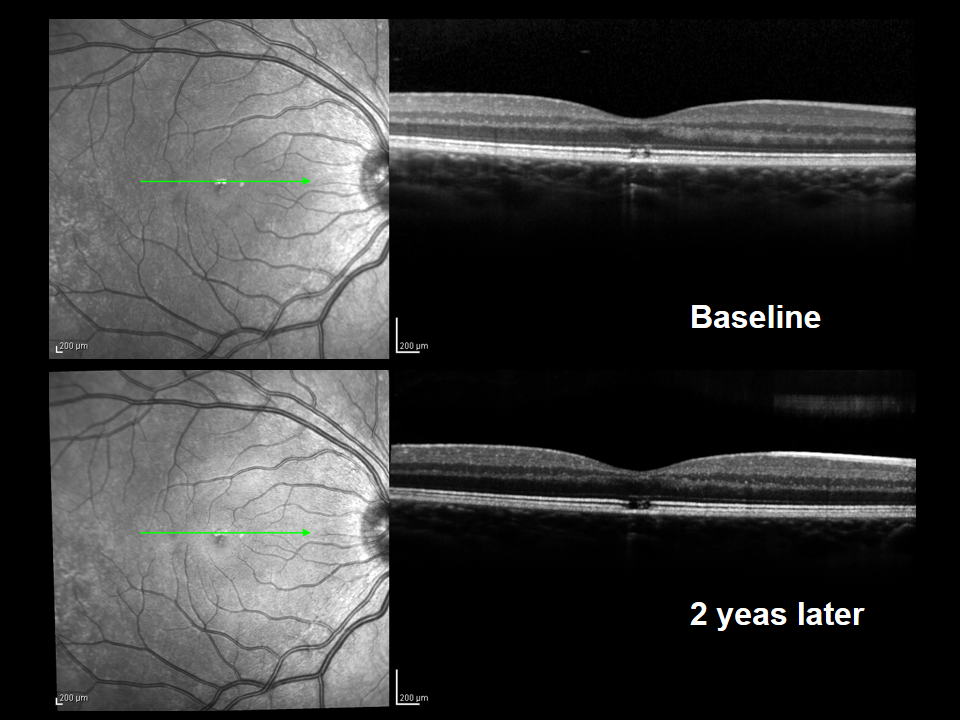

Supplement: Supplementary file 3 — Supplementary image 3 [file 41433_2018_276_MOESM3_ESM.tif]
