# Supplementary material for: Retinal burns from laser pointers: a risk in children with behavioural problems
Source: Eye (Lond). 2018 Dec 13;33(3):492–504. doi: 10.1038/s41433-018-0276-z (PMC6460723; doi:10.1038/s41433-018-0276-z)
Supplement: Supplementary file 4 — LEGENDS FOR SUPPLEMENTARY FIGURES [file 41433_2018_276_MOESM4_ESM.docx]

**Retinal burns from laser pointers explored using mixed methods. Novel risk in**

**children with behavioural problems highlighted with implications for policy.**

**LEGENDS FOR SUPPLEMENTARY FIGURES**

**SUPPLEMENTARY IMAGE 1**. Patient 1: right eye. Infrared and linked OCT

images at baseline visit on top panel and following 2 years on lower panel.

**SUPPLEMENTARY IMAGE 2.** Laser pointer as was retrieved from Patient 3.

**SUPPLEMENTARY IMAGE 3.** Patient 3. Right eye. Baseline and year 2 linked

infrared and OCT images. The outer lamellar layer defects persist.
